# 12-Month peak alpha frequency is a correlate but not a longitudinal predictor of non-verbal cognitive abilities in infants at low and high risk for autism spectrum disorder

**DOI:** 10.1016/j.dcn.2021.100938

**Published:** 2021-03-03

**Authors:** Virginia Carter Leno, Andrew Pickles, Stefon van Noordt, Scott Huberty, James Desjardins, Sara Jane Webb, Mayada Elsabbagh, The BASIS Team

**Affiliations:** a Institute of Psychiatry, Psychology & Neuroscience, King’s College London, UK; b Montreal Neurological Institute, Azrieli Centre for Autism Research, McGill University, Montréal, Canada; c Compute Ontario, Canada; d Center on Child Health, Behavior and Development, Seattle Children’s Research Institute, Seattle, WA, USA

**Keywords:** Autism spectrum disorder, Peak alpha frequency, Cognitive development, EEG, Infant siblings

## Abstract

Although studies of PAF in individuals with autism spectrum disorder (ASD) report group differences and associations with non-verbal cognitive ability, it is not known how PAF relates to familial risk for ASD, and whether similar associations with cognition in are present in infancy. Using a large multi-site prospective longitudinal dataset of infants with low and high familial risk for ASD, metrics of PAF at 12 months were extracted and growth curves estimated for cognitive development between 12–36 months. Analyses tested whether PAF 1) differs between low and high risk infants, 2) is associated with concurrent non-verbal/verbal cognitive ability and 3) predicts developmental change in non-verbal/verbal ability. Moderation of associations between PAF and cognitive ability by familial risk status was also tested. No differences in 12-month PAF were found between low and high risk infants. PAF was associated with concurrent non-verbal cognitive ability, but did not predict change in non-verbal cognitive over development. No associations were found between PAF and verbal ability, along with no evidence of moderation. PAF is not related to familial risk for ASD, and is a neural marker of concurrent non-verbal cognitive ability, but not verbal ability, in young infants at low and high risk for ASD

## Highlights

• Peak alpha frequency (PAF) is thought to be a more sensitive and reliable measure of alpha band oscillatory activity.
• Higher PAF has been associated with better performance on a range of cognitive tasks.
• This study examines associations between PAF and non-verbal and verbal cognitive development in young infants aged 12–36 months.

## 1 Introduction

Oscillations in the alpha frequency band (typically 8−12 Hz in adults) are the dominant rhythm captured by scalp recorded electroencephalography (EEG), and thus often studied in relation to psychiatric disorders, including autism spectrum disorder (ASD) (Newson and Thiagarajan, 2019). Alpha rhythms are thought to play an important role in cognitive functioning by modulating the degree and timing of cortical inhibition and communication (Chapeton et al., 2019; Klimesch, 1999; Klimesch et al., 2007). However, findings with regard to differences in alpha power in autistic individuals are mixed. Research into the neurobiological basis of ASD has reported decreases (Cantor et al., 1986; G. Dawson et al., 1995; Shephard et al., 2018), increases (Cornew et al., 2012) and no difference (Coben et al., 2008; Lefebvre et al., 2018) in alpha power in autistic individuals as compared to typically developing individuals. In addition to the more general sources of variation between samples and methodological approaches (see O’Reilly et al., 2017 for details), the lack of precision of in averaging across the alpha band may explain the lack of agreement among previous studies.

In adults, alpha power is measured as the amount of power (μV^2^/Hz) between the boundaries of 8−12 Hz. Prior work suggests that “alpha” is represented by a lower frequency range in early development and doesn’t become adult like until around age 11 (Samson-Dollfus et al., 1997).

In addition to the impact of developmental maturation on where exactly “alpha” is located, studies of alpha power in adults find large inter- and modest intra-individual differences in the peak of alpha oscillations (Haegens et al., 2014). This variability may lead neighboring frequency band oscillations (e.g., theta, beta) to be included, and relevant alpha oscillations to not be included, in the calculation of summed alpha power. Peak alpha frequency (PAF), the frequency at which oscillations in the alpha range demonstrate maximum power, may be a more sensitive and reliable measure of alpha band oscillatory development (Levin et al., 2020) and has been argued to be a more precise measure of the magnitude of alpha oscillatory activity (Haegens et al., 2014). PAF is not a static phenomenon over development – it has been shown to increase from infancy through childhood (Marshall et al., 2002; Miskovic et al., 2015), likely a reflection of the increasing complexity of cortical organisation over time. In addition, in typically developing individuals, higher PAF is associated with better performance on a range of cognitive tasks (for a review see Klimesch, 1999) and higher standardized IQ tests (Anokhin and Vogel, 1996). PAF is also related to neurodevelopmental delay (Dickinson et al., 2018; Edgar et al., 2019). One study reported higher PAF in autistic individuals (Edgar et al., 2019), but this effect appeared to be isolated to only the younger participants (<10 years old). Others report lower PAF in autistic vs. typically developing children (mean age 5 years) (Dickinson et al., 2018), or no differences (mean age 9 years) (Lefebvre et al., 2018). PAF at 12 and 24-month old in infants with tuberous sclerosis complex (TSC) did not differ on the basis of an additional diagnosis of ASD (Dickinson et al., 2019). Extant literature also suggests a link between PAF and cognitive functioning in autistic populations (Dickinson et al., 2018, 2019; Edgar et al., 2019), similar to that found in typically developing groups (Klimesch, 1999). Given the cross-sectional associations between PAF and non-verbal ability in autistic individuals, PAF has been suggested as a potential predictor of later outcomes. This would be advantageous as it could be used to delineate autistic children who may be more likely to have later difficulties in cognitive functioning, thus allowing interventions to be offered to those most in need early in the developmental pathway (Kaaresen et al., 2008; Infant Health and Development Program, 1990). Thus, it is key to understand whether associations between PAF and relevant aspects of the autistic phenotype (e.g., cognitive ability) reported in autistic children and adults are also present in infancy. The viability of PAF as a predictor of future cognitive functioning also depends on whether it is associated with prospective change in non-verbal cognitive ability, which has yet to be established. In addition to the clear benefits of identifying markers of later outcomes in autistic individuals, establishing longitudinal associations is a key step in building a mechanistic model of the heterogeneity in cognitive outcomes in autistic individuals. Finally, although limited, there is evidence to suggest associations between PAF and non-verbal ability might be group specific. Dickinson et al. (2018) found a positive association between PAF and non-verbal IQ in autistic individuals, but no association in typically developing individuals. In the typically developing group PAF was predicted instead by age. Similarly, Edgar et al. (2019) found the association between PAF and non-verbal IQ was only present in the older (>10 years old) autistic individuals in their study. Although three studies have looked at how PAF relates to ASD diagnosis, none have yet tested how PAF relates to heritable risk for ASD (typically studied in infants who have an older sibling with a diagnosis). Knowledge of how PAF relates to familial ASD risk is required to determine if it could be a marker of aberrant cortical development before the behavioural symptoms of ASD emerge. This is especially relevant to PAF given its high heritability (Smit et al., 2005), making it a potential candidate to explore one mechanism by which heritable risk translates to differences in early neural and cognitive functioning.

### 1.1 Current study

We use a pooled repository of EEG and cognitive data to give adequate statistical power, and take advantage of repeated measurements of cognitive functioning to estimate developmental trajectories of non-verbal/verbal cognitive ability over infancy. We test 1) whether differences in PAF are found between infants at low vs. high familial risk for developing ASD 2) whether PAF predicts both concurrent and/or developmental change in non-verbal ability and 3) whether associations between PAF and non-verbal ability are moderated by familial risk status. We also test the same associations with verbal ability as the outcome to assess specificity of findings.

## 2 Method

### 2.1 Sample

We used data from the International Infant EEG Platform (EEG-IP; van Noordt et al., 2020). This data integration platform combines data from previous longitudinal infant sibling studies run by Birkbeck University London and the University of Washington in Seattle (for more details on sample breakdown and inclusion criteria see Huberty et al., under review). All samples within the repository were collected in accordance with the ethical standards of the institutional and/or national research committee and with the 1964 Helsinki Declaration and its later amendments or comparable ethical standards. Parents The full sample comprised 196 infants (n = 106 from London, n = 90 from Seattle). Infants were designated as being at low or high familial risk for ASD by virtue of either having an older sibling diagnosed with ASD or no family history of ASD; 49 % (n = 95) were at low risk and 51 % (n = 97) were at high risk (4 were missing information about risk status). ASD outcome (present/absent) was assessed by gold-standard diagnostic instruments (e.g., the Autism Diagnostic Observation Schedule (ADOS); Lord et al., 2000), administered at 24 and/or 36 months, along with clinical judgement; 17 % (n = 32) of the sample met diagnostic criteria for ASD. There were no site differences in the proportion at low/high risk (χ^2^ = .18, p = .67) or who did/did not receive a diagnosis of ASD (χ^2^ = .16, p = .69). We present information on data points available for the measures used in the current analyses in Table 1. Of the full 196, 151 had available phenotypic data and usable 12-month EEG data (see below for pre-processing steps).

**Table 1:** Data Availability and Sample Characteristics.

	Whole Sample		Low Risk	High Risk
	N (London/ Washington)	Mean (SD; range)	Mean (SD; range)	Mean (SD; range)
12 month visit	182 (100/82)			
Age at visit		13.03 (1.43; 11−18)	13.08 (1.41; 11−17)	12.98 (1.45; 11−18)
MSEL non-verbal AE		15.47 (1.98; 8.5−23)	16.03 (1.79; 16−24.5)	14.96 (2.01; 8.5−23)
MSEL verbal AE		12.51 (2.61; 7.5−22)	12.97 (2.33; 9−22)	12.08 (2.80; 7.5−22)
Peak alpha frequency (Hz)		7.45 (0.44; 6.35−8.88)	7.44 (0.47; 6.35−8.88)	7.46 (0.43; 6.61−8.87)

18 month visit	78 (0/78)			
Age at visit		18.10 (0.52; 17−20)	18.10 (0.60; 17−20)	18.10 (0.45; 17−20)
MSEL non-verbal AE		19.53 (1.88; 15.5−24.5)	19.82 (1.75; 16−24.5)	19.23 (1.98; 15.5−24)
MSEL verbal AE		17.89 (3.78; 7.5−27)	18.14 (3.22; 10−24)	17.64 (4.28; 7.5−27)

24 month visit	138 (95/43)			
Age at visit		24.04 (1.00; 21−28)	23.88 (0.66; 23−26)	24.13 (1.14; 21−28)
MSEL non-verbal AE		25.69 (3.45; 17.5−36.5)	26.80 (3.61; 18.5−36.5)	25.12 (3.23; 17.5−34)
MSEL verbal AE		25.86 (5.32; 11.5−39.5)	28.09 (4.20; 17−39.5)	24.70 (5.49; 11.5−34.5)

36 month visit	100 (100/0)			
Age at visit		37.93 (3.02; 32−53)	38.23 (3.05; 25−51)	37.66 (2.99; 32−53)
MSEL non-verbal AE		41.07 (6.65; 21−59.5)	42.80 (5.06; 27.5−55.5)	39.46 (7.54; 21−59.5)
MSEL verbal AE		41.44 (8.39; 8.5−58.5)	44.11 (6.68; 31.5−58.5)	39.02 (9.08; 8.5−56.5)

AE = age equivalent, MSEL = Mullen Scales of Early Learning, SD = standard deviation.

### 2.2 Measures

#### 2.2.1 Verbal and non-verbal ability

The Mullen Scales of Early Learning (MSEL; Mullen, 1995) is a standardized assessment that is commonly used to measure cognitive development in young infants. The MSEL is organized into 5 subscales: (a) gross motor, (b) fine motor, (c) visual reception (or non-verbal problem solving), (d) receptive language, and (e) expressive language. Each subscale is standardized to calculate a t-score, standard score, percentile and age-equivalent score. Non-verbal ability was estimated using the average of the age-equivalent scores from the visual reception and fine motor subscales, verbal ability using the average of the age-equivalent scores from the receptive and expressive language subscales.

#### 2.2.2 EEG

##### 2.2.2.1 EEG data collection

At both sites in EEG-IP, high-density scalp EEG was recorded continuously using a 128-channel HydroCel Geodesic Sensor Net system (Electrical Geodesics, Eugene, OR). EEG data were collected while infants watched videos on a monitor sitting on their caregiver’s lap in a dark room. The Seattle sample videos consisted of a set of moving toys with sound, and a set of an adult woman facing the camera and singing nursery rhymes. The London sample consisted of the same videos, and there was an additional third set of videos of moving toys being activated by a human hand. The two video sets in the Seattle sample lasted 60 s each and the three video sets in the London sample lasted 30−40 s each.

##### 2.2.2.2 EEG pre-processing

In EEG-IP, raw data contributed by each site was harmonized and standardized in order to be maximally compatible. Several open source solutions were employed, including use of the Brain Imaging Data Structure extension to EEG (Pernet et al., 2019) and standardized pre-processing using the Lossless Pipeline (https://github.com/BUCANL/bids_lossless_eeg; Desjardins et al., under review). Briefly, the pre-processing involved a set of automated procedures to identify unreliable signals and non-stationarity in scalp channels and independent components and their time courses. Comprehensive data annotation was integrated with the raw EEG signals for expert quality control review. Further details of pre-processing and quality control procedures in EEG-IP are provided (Desjardins et al., under review; van Noordt et al., 2020). Although EEG data were available from 155 infants at 12-months, two participants had no data on non-verbal/verbal cognitive or risk status so were excluded prior to EEG pre-processing. Participants who had <8 epochs after data cleaning were excluded (n = 2), leaving a total of 151 participants with usable EEG data (n = 83 from London, n = 68 from Seattle).

##### 2.2.2.3 Spectral decomposition

EEG channels were interpolated to correspond to the 10–20 system (F7, Fpz, AF8, F3, Fz, F4, FT7, C3, Cz, C4, FT8, TP7, P3, Pz, P4, TP8, PO7, Oz, PO8). Data were segmented into 4000 ms 50 % overlapping epochs. For each electrode, power spectral density (PSD) of the area under the curve (trapezoidal numerical integration) was computed with the Welch method using the *pwelch* function in MATLAB. A Hamming window was applied before estimating a modified periodogram with a 0.25hz frequency bin resolution (4 s) for each segment. Periodograms for all segments were averaged to produce a final spectral estimate. Electrode-level estimates of PSD were averaged to generate frontal (F3, F4, Fz), central (C3, C4, Cz), parietal (P3, P4, Pz) and occipital (PO7, PO8, Oz) ROIs.

##### 2.2.2.4 Extraction of PAF

**Fig. 1:**
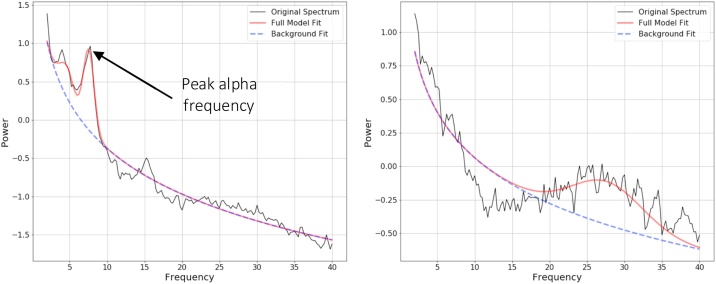
Illustration of the parameters extracted by the FOOOF algorithm. The figure on the left demonstrates an example of appropriate alpha peak identification, whereas the figure on the right demonstrates an example of a participant who had no clear alpha peak. Power values are log transformed.

The fitting oscillations and one over f (FOOOF) algorithm was used to parameterize neural power spectra and obtain individual PAF values (Donoghue et al., 2020). The FOOOF algorithm first estimates and removes the aperiodic ‘background’ slope (the dotted background line in Fig. 1) from the absolute PSD. After removing the aperiodic slope component, the remaining periodic oscillatory peaks are modelled as individual Gaussian curves. Each oscillatory peak is characterised by its own amplitude, centre frequency, and bandwidth. This algorithm has been shown to extract reliable estimates from populations of youth with ASD (Levin et al., 2020), and allows one to extract estimates of alpha oscillations that take account of variation in aperiodic background activity. Input parameters for the algorithm were set as: peak width limits: 1.0–10.0; max number of peaks: 3; minimum peak height: 0.4; peak threshold: 1.85; and aperiodic mode: fixed. These settings were chosen by visually inspecting model fit across a range of parameters (blind to risk group or outcome) and selecting those that gave a model that best matched the original raw PSDs without overfitting (see Fig. 1 for examples of FOOOF outputs with and without a clear alpha peak).

**Fig. 2:**
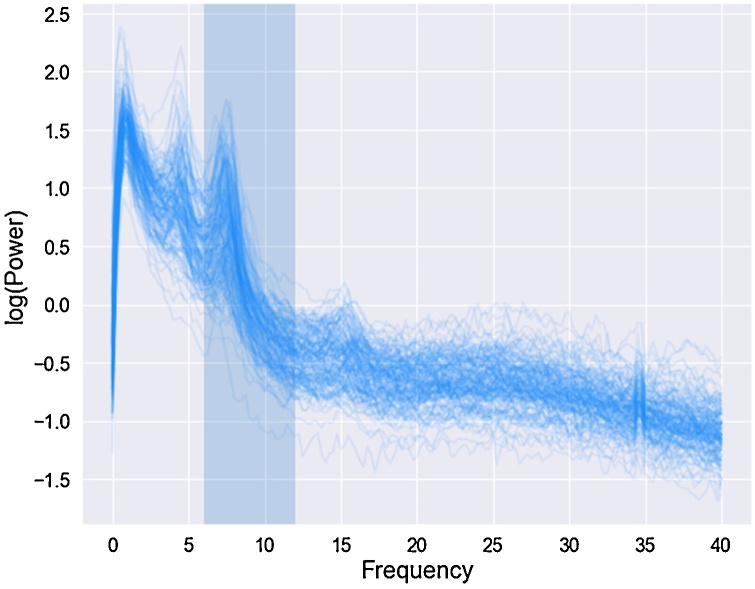
12-month power spectral density’s (PSD) at 0-40 Hz for all participants at central (C3, C4, Cz) region of interest. Peak alpha frequencies were extracted from the highlighted frequency range (6−12 Hz). Power values are log transformed.

Power spectra were parameterized across the frequency range 2–40 Hz. Analyses focused on PAF extracted from the central ROI as it had the highest number of participants with an identifiable peak in the 6−12 Hz range (127/151, 84 %); this corresponds with previous reports of reliable identification of the alpha rhythm across central ROIs in infancy (Marshall et al., 2002) and the topographic location of ASD case-control differences (Dickinson et al., 2018, 2019). Only those with an identifiable peak in the 6−12 Hz range could be included in the analyses; we selected this frequency range based on previous developmental work suggesting 6 Hz is an appropriate lower bound for the alpha rhythm in infants from 5 months upwards (Marshall et al., 2002). Fig. 2 displays PSDs for the whole sample to illustrate there was a clear peak in the 6−12 Hz range. There were five participants where FOOOF identified two peaks in 6−12 Hz range in the central ROI; in these participants the higher value was taken as their PAF. There were no significant differences in the proportion of participants who had an identifiable peak by site (χ^2^ = .28, p = .59) or risk status (χ^2^ = .62, p = .43).

### 2.3 Statistical analysis

**Fig. 3:**
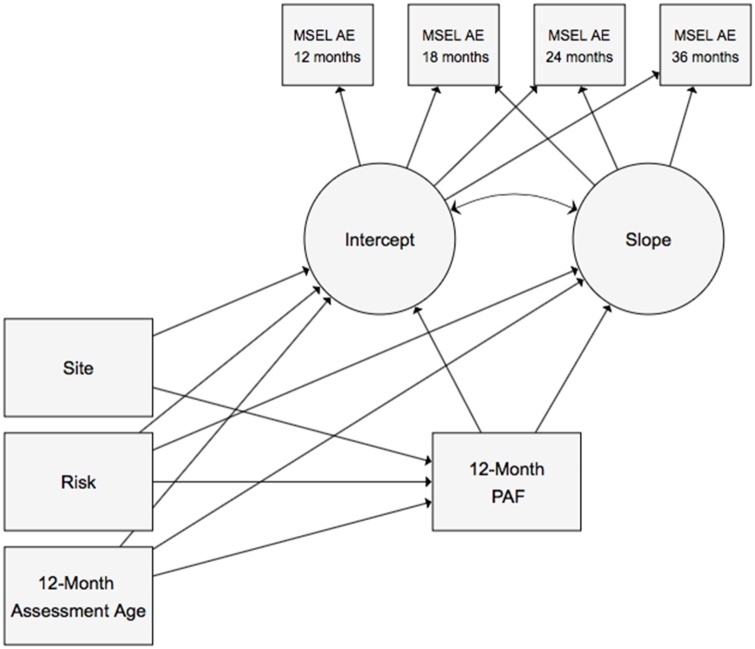
Growth Curve Model to Test Concurrent and Longitudinal Associations between Peak Alpha Frequency (PAF) and Cognitive Development.

All analyses were conducted in Stata 14. First, we used an analysis of variance (ANOVA) to test whether 12-month PAF differed by familial risk (low vs. high), whilst adjusting for age at 12-month assessment and site (London vs. Seattle). Next, a latent growth curve model (GCM) was fit to the non-verbal/verbal AE data using the sem command. Intercept (the estimated non-verbal/verbal ability at 12 months) and slope (the estimated change in non-verbal/verbal ability over time) were specified as latent variables, which varied across individuals. We then tested whether 12-month PAF significantly predicted concurrent non-verbal/verbal ability (the intercept), and the rate of non-verbal/verbal ability development from 12 to 36 months (the slope), while controlling for age at 12-month assessment, familial risk and site (see Fig. 3 for a diagram of the model with non-verbal ability as the outcome). We tested whether there were any differences in the association between 12-month PAF and non-verbal/verbal ability intercept or slope by risk status by including a PAF-by-risk interaction term as an additional predictor. We also assessed the impact of infants who went onto receive a diagnosis of ASD in toddlerhood by excluding them and re-running longitudinal analyses (the group was not large enough to run robust moderation analyses in the same manner as with risk status). To assess the impact of the 11 infants who were aged >40 months at their 36-month visit (7 low risk, 7 high risk) upon results, analyses were re-run without these participants (Supplementary Materials). All models were estimated using method (mlmv) to account for missing data under the “missing at random” assumption, under which missingness is assumed to relate only to observed variables in the model. The inclusion of site accounts for the differing measurement schedules at each with data missing by design. Wald tests were used to assess the significance of paths. All reported path coefficients are unstandardized coefficients.

## 3 Results

### 3.1 Sample characteristics

See Table 1 for sample means of PAF, age at 12-month visit and non-verbal/verbal ability age-equivalent scores between 12 and 36 months. In addition to the information displayed in Table 1, we assessed site differences before running growth curve models. The mean age at the 12 month visit differed by site, with infants completing assessments at an older age in London compared to Seattle (13.79 months vs. 12.10 months; t(181) = 9.86, p < .01). A trend was observed for site differences in PAF (t(125) = 1.85, p = .07), with an average PAF of 7.52 Hz in London as compared to 7.37 Hz in Washington.

### 3.2 PAF and risk status

**Fig. 4:**
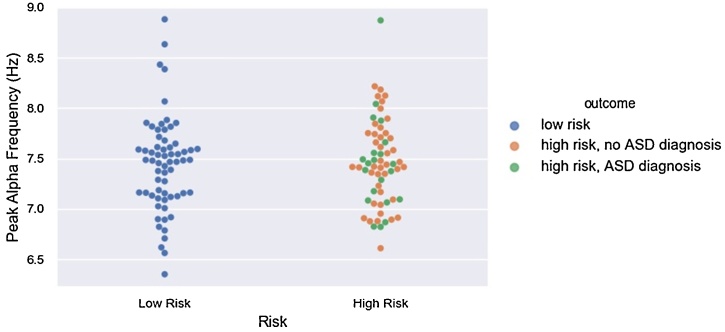
No Difference in 12-Month Peak Alpha Frequency by Familial Risk Status. Although group comparisons were only made on the basis of risk, both risk and outcome groups are depicted in the figure to aid interpretation

Analyses did not show differences in 12-month PAF between infants with low vs. high familial risk for ASD (F(1, 125) = .01, p = .94) (see Fig. 4). The mean PAF in the low risk group was 7.44 Hz (range = 6.35−8.88 Hz), and the mean PAF in the high risk group was =7.46 Hz (.43, range = 6.61−8.87 Hz).

### 3.3 PAF and non-verbal ability

#### 3.3.1 Predictors of 12-month PAF

12-month PAF was significantly associated with age at the 12-month visit (b = .08, 95 % CIs = .02 − .14, p < .01), but not risk (b = .03, 95 % CIs = -.12 − .18, p = .68), nor site (b =-.01, 95 % CIs = -.19 − .18, p = .96).

#### 3.3.2 Predictors of intercept

**Fig. 5:**
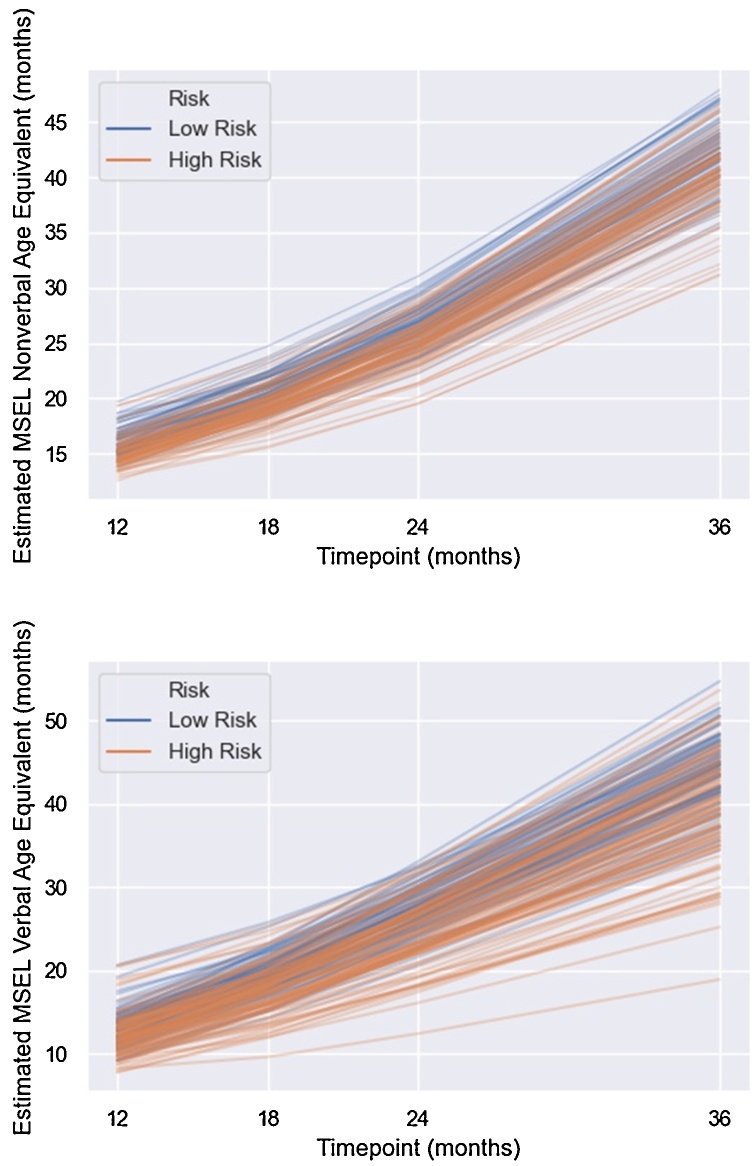
Estimated Growth in Non-Verbal (top) and Verbal Ability (bottom) Between 12-36 Months in Infants at Low and High Familial Risk for ASD.

Risk (b= -.95, 95 % CIs = −1.39 to −.50, p < .01), age at the 12-month visit (b = .56, 95 % CIs = .36 − .76, p < .01) and 12-month PAF (b = .84, 95 % CIs = .22–1.46, p = .01), all predicted non-verbal ability intercept, with being in the low risk group, being older and having a higher PAF all associated with higher non-verbal ability at the 12-month visit (see Fig. 5 for individual trajectories of growth in non-verbal ability, split by risk group). Site was not a significant predictor of intercept (b= -.18, 95 % CIs = -.75 − .39, p = .54).

#### 3.3.3 Predictors of slope

Age at 12-month visit predicted slope (b= -.20, 95 % CIs = −.35 to −.06, p < .01) such that participants who were older at the 12 month visit had less steep slopes of change in non-verbal ability between 12–36 months. Neither 12-month PAF (b= -.22, 95 % CIs = -.73 − .30, p = .41) nor risk (b= -.24, 95 % CIs = -.64 − .16,p = .25) were significant predictors (see Fig. 5).

#### 3.3.4 Moderation by risk

**Fig. 6:**
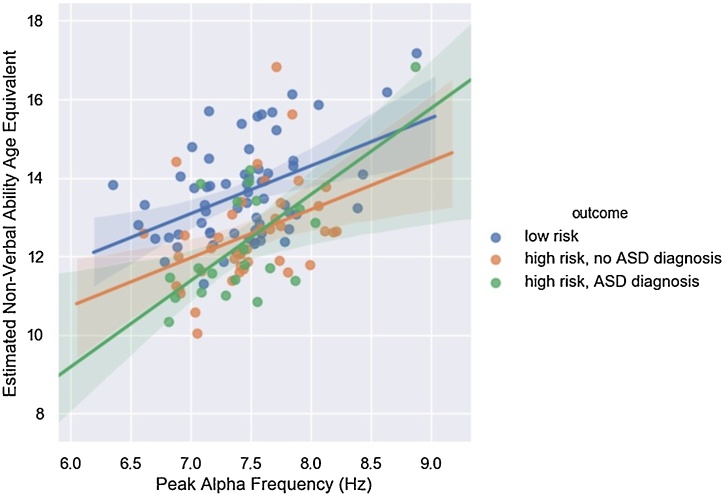
Positive Association Between 12-Month Peak Alpha Frequency and Estimated Non-Verbal Ability Intercept. Although the model was only run with risk, both risk and outcome groups are depicted in the figure to aid interpretation. Lines represents the best fitting regression line for each group, shaded areas represent 95 % confidence intervals.

The 12-month PAF-by-risk interaction term was not significant in the prediction of non-verbal ability intercept (b = 1.12, 95 % CIs = -.16 – 2.40, p = .09) or slope (b= -.04, 95 % CIs = -.98 − .89, p = .93), suggesting associations between 12-month PAF and non-verbal ability were comparable between low and high risk infants (see Fig. 6). When infants who went on to receive an ASD diagnosis in toddlerhood were excluded from the analyses (n = 22), risk and age at 12-month visit remained predictors of non-verbal ability intercept (b= -.71, 95 % CIs = −1.17 to −.24, b = .60, 95 % CIs = .39 − .82, respectively, both ps<.01) but the association between 12-month PAF and non-verbal ability intercept became non-significant (b = .45, 95 % CIs = -.21 – 1.11, p = .18).

### 3.4 PAF and verbal ability

#### 3.4.1 Predictors of intercept

Similar to the non-verbal ability models, risk (b= -.82, 95 % CIs = −1.49 to −.16, p = .02) and age at the 12-month visit (b = .70, 95 % CIs = .40 − 1.00, p < .01) both predicted verbal ability intercept, such that high-risk and younger participants had lower verbal ability scores at 12 months (see Fig. 5). Neither 12-month PAF (b = .69, 95 % CIs = -.28 – 1.66, p = .16) nor site significantly predicted intercept (b= -.35, 95 % CIs = -1.18 − .47, p = .40).

#### 3.4.2 Predictors of slope

Age at the 12-month visit (b= -.40, 95 % CIs = −.63 to −.18, p < .01), and risk predicted slope (b= -.92, 95 % CIs = −1.53 to −.31, p < .01), such that infants who were older and those that were high risk had a less steep slope of verbal ability change between 12 and 36 months (see Fig. 5). PAF did not predict slope (b=-.16, 95 % CIs = -.96 − .65, p = .70).

#### 3.4.3 Moderation by risk

The 12-month PAF-by-risk interaction term was not significant in the prediction of verbal ability intercept (b= -.63, 95 % CIs = -2.58 – 1.33, p = .53) or slope (b = .47, 95 % CIs = -1.04 – 1.98, p = .55), suggesting associations between 12-month PAF and verbal ability were comparable between low and high risk infants.

When infants who went on to receive an ASD diagnosis in toddlerhood were excluded, only age at the 12-month visit remained a predictor of verbal ability intercept (b = .89, 95 % CIs = .57–1.22, p < .01); however, both risk and age at the 12-month visit predicted verbal ability slope (b= -.82, 95 % CIs = -1.39 – -.25; b= -.45, 95 % CIs = -.66 – -.24, both p < .01) such that older participants and those with high risk had less steep slopes of change in verbal ability between 12–36 months.

## 4 Discussion

The current study had three aims. First, to test for differences in PAF between 12-month old infants at low vs. high familial risk for developing ASD. Second, to test whether PAF measured at 12 months was associated with non-verbal cognitive ability, both concurrently and longitudinally. Third, to test whether associations between PAF and non-verbal cognitive ability were moderated by familial risk status, and to what extent infants that went onto receive a diagnosis of ASD in toddlerhood were contributing to any significant PAF- non-verbal cognitive ability associations. We examined the same longitudinal relationships with verbal ability as the outcome to assess the specificity of associations between PAF and non-verbal vs. verbal abilities. Analyses showed there were no differences in 12-month PAF depending on familial risk status. As hypothesised, we found a positive association between PAF and concurrent non-verbal cognitive ability, replicating previous findings from typically developing infants and infants with TSC (Dickinson et al., 2019). However, we did not find evidence that PAF predicted the development of non-verbal ability between 12–36 months. Results showed no strong evidence for moderation of the association between PAF and non-verbal cognitive ability by risk status, although we note that the interaction term for the association between PAF and concurrent non-verbal cognitive ability was marginal. We found no significant associations between PAF and verbal ability intercept or slope, suggesting associations with concurrent PAF may be specific to non-verbal cognitive abilities.

In the current sample of 12-month old infants, the average PAF was 7.45 Hz. This is in line with previous reports of infant alpha oscillations that also broadly find central PAF to be around 7 Hz in the first year of life (Marshall et al., 2002; Stroganova et al., 1999). Across both growth curve models, age at the 12-month visit predicted 12-month PAF. This was to be expected given the reported increase in PAF across development, but it is of interest that increasing age was associated with increasing PAF in a relatively narrow window (11−18 months). This finding also reinforces the importance of adjusting for age when testing associations between PAF and non-verbal/verbal cognitive ability, as it is associated with both variables. The lack of difference in PAF between infants at low vs. high familial risk for ASD is contrast to two studies that have found differences in PAF in autistic children as compared to a typically developing group; one reporting higher PAF (Edgar et al., 2019) and the other reporting lower PAF (Dickinson et al., 2018) in the autistic group (although we highlight that only a small subgroup of the high risk infants will go onto receive a diagnosis of ASD). However, two studies have also found no group differences; one with infants with TSC, with and without an additional diagnosis of ASD (Dickinson et al., 2019), and another case-control study in children (Lefebvre et al., 2018). Therefore, results suggest that disrupted PAF is not an early manifestation of familial autism risk. An alternative explanation is that PAF development may be slowed in infants who go on to receive a diagnosis such that clear group differences only become apparent as neural trajectories diverge later in development. Clarification of these two proposed hypotheses requires following large, well-characterised samples of autistic individuals with multiple measurements of neural functioning from early in childhood.

In line with previous work in both autistic and typically developing samples (Dickinson et al., 2018; Doppelmayr et al., 2002; Klimesch, 1999), we found a positive association between 12-month PAF and concurrent non-verbal cognitive ability, even when adjusting for age. Furthermore, results suggest this association is relatively specific; no associations were found between 12-month PAF and verbal ability (either concurrently or longitudinally). Given that PAF is associated with both functional and structural network connectivity (Jann et al., 2012), and oscillations in the alpha band modulate cortical inhibition (Klimesch et al., 2007), this would suggest higher cognitive abilities at 12 months are supported by more mature cortical organisation and more effective communication. This is of interest as it appears these associations between brain and cognition are present in some of the earliest stages of development; most existing literature has focused on associations with PAF in older children and adults. It may be that PAF is a stable marker of non-verbal cognitive ability across the lifespan, which suggests it could be of use as an additional metric of cognitive functioning in samples with wide ranges of biological or developmental age, which traditionally use multiple instruments to measure IQ depending on participant age and ability. This would be advantageous, as it would provide a metric of cognitive ability that could easily be compared across participants in heterogeneous samples while at the same time minimise biases that come with face-to-face cognitive assessments. The use of in-person cognitive tests (such as that included in the present study) in autistic populations has been criticised for a variety of reasons, such as over-reliance on language, sustained attention and social compliance (Kenworthy et al., 2008). These domains are ones where autistic individuals may have particular difficulty, which may explain reports of underestimation of ability when traditional cognitive tests are used in autistic populations (M. Dawson et al., 2007). The proposal of PAF as a marker for non-verbal cognitive ability is further strengthened by the lack of association with verbal ability, suggesting it is a relatively specific marker of cognitive abilities that are independent from language or other verbal components of cognitive functioning. As a caveat to this last point, we acknowledge that the lack of association with verbal ability could also be due to increased measurement error in assessing VIQ as compared to non-verbal cognitive ability, especially in populations known to have atypical styles of communication. Furthermore, it should be noted that we did not directly test for differential associations, but included verbal ability as an outcome to assess whether there was any evidence for specificity.

The association between PAF and non-verbal cognitive ability appeared to be comparable between infants at low vs. high risk, however as noted above, the interaction term was at a marginal level of significance in predicting the non-verbal ability intercept (p = .09), and the directionality of effect was the same as in previous work which has focused on individuals with an established diagnosis of ASD (Dickinson et al., 2018; Edgar et al., 2019), in that the association was significant in high but not low risk infants. When we excluded the subgroup of infants who went onto receive a diagnosis of ASD in toddlerhood, associations between PAF and concurrent non-verbal cognitive ability were no longer significant. This suggests that this subgroup of infants may have been making a substantial contribution to the association between PAF and concurrent non-verbal cognitive ability found in the whole sample. Comparing our results to samples of older individuals with an established diagnosis (Dickinson et al., 2018; Edgar et al., 2019), one could conclude PAF is only a marker for non-verbal cognitive abilities in individuals who have or will have a diagnosis of ASD. A more prosaic explanation is that there is simply less variability in cognitive ability in typically developing infants and children, meaning significant associations with PAF are less likely to be detected in typically developing populations.

Contrary to extant literature (Dickinson et al., 2019), current results suggest PAF may not be a suitable prognostic marker for future trajectories of cognitive development. There are several possible interpretations of our finding of no prospective association between PAF and non-verbal cognitive ability. The most obvious is that PAF is a marker of current cognitive functioning, but does not predict change in cognitive development. It may be that a more direct measure of cortical maturation (e.g., connectivity; Xie et al., 2019) is needed to predict change in cognitive development over time, rather than proxy markers such as PAF. Two counter arguments should be considered. First, is that our current tools to assess cognitive functioning in infants do not adequately capture subtle developmental changes in cognitive ability. This may be especially the case in infants who are more likely to have atypical behaviours due to increased familial risk. Second, that the sample included at present may not be population-representative; indeed the sample had an average non-verbal AE at 12 months of 15.5, suggesting the sample as a whole was scoring above what is to be expected at their age. Therefore, it is possible the sample (especially the low risk group) had less variability in their cognitive development due to unaccounted for sampling biases, which in turn would decrease power to detect associations between PAF and slope of change. We note this issue of “super-healthy controls” is not specific to infant-sibling studies, but psychiatric research in general (Lee et al., 2007).

The present study has many strengths. We take advantage of the newly developed EEG-IP repository, meaning our analyses were conducted on a sample substantially larger than most studies in the field. We extracted PAF, which is thought to be a more sensitive marker of individual differences in alpha band oscillatory activity than traditional power metrics. We also utilised data from infants followed longitudinally over multiple visits, allowing us to model rates of cognitive development. This meant we could build on previous work by examining how PAF relates to rate of change in cognitive abilities rather than just ability at a specific time point. This gives a more nuanced understanding of how different trajectories of development may underpin atypical outcomes that are often associated with neurodevelopmental disorders. The current study also has limitations; by design all infants came from families with at least one existing autistic child, thus whether PAF functions in a similar manner in cases where no family history of ASD is present, and whether brain-behaviour associations depend on the level of familial risk (e.g., number of existing autistic siblings) requires further investigation. This issue is of particular importance given reports that autistic infants differ on cognitive ability depending on heritable genetic load (Dissanayake et al., 2019; McDonald et al., 2020).

Current results support the suggestion that PAF is a robust marker of non-verbal cognitive ability, even in early infancy, and may be a useful metric of non-verbal cognitive ability to complement in-person standardized IQ tests. We found no associations with verbal ability, which can be interpreted two ways. Either PAF may be a fairly specific marker of non-verbal rather than verbal abilities (e.g., language), or that current measures to assess verbal abilities in infants (especially those at risk for ASD) have poor sensitivity. We did not find evidence that PAF predicts the development of non-verbal cognitive ability through infancy, suggesting it may not be an appropriate predictor of outcomes. Given the heterogeneity in outcomes for infants who go onto receive a diagnosis of ASD, future work should test if more proximal metrics of cortical development predict outcomes across a variety of domains. This would allow identification of those who may need additional support, and promote positive long-term outcomes.

## Data statement

Data sharing agreements with data contributors do not offer the option of sharing data at this current time.

## Declaration of Competing Interest

The authors report no declarations of interest.
