# Supplementary material for: 12-Month peak alpha frequency is a correlate but not a longitudinal predictor of non-verbal cognitive abilities in infants at low and high risk for autism spectrum disorder
Source: Dev Cogn Neurosci. 2021 Mar 3;48:100938. doi: 10.1016/j.dcn.2021.100938 (PMC7966984; doi:10.1016/j.dcn.2021.100938)
Supplement: Supplementary file 1 [file mmc1.docx]

**Supplementary Materials**

**Sensitivity analyses excluding the eleven infants who were >40 months of age at the 36-month assessment**

The final model with 12-month PAF, site, risk and age at 12-month visit as predictors of MSEL intercept and slope would not converge, likely because all the excluded infants came from the London sample. Therefore we re-ran the models without site, including and excluding said infants, and report standardised estimates to aid comparability between the two. As the main focus was whether these infants could be contributing to significant results, we only report results for the association between 12-month PAF and non-verbal/verbal ability intercept and slope.

*Non-Verbal Ability*

In the full sample, the standardised coefficient between 12-month PAF and non-verbal ability intercept was β=.29 (95% CIs = .06 - .51, p=.01). The standardised coefficient between 12-month PAF and non-verbal ability slope was β=-.14 (95% CIs = -.49 - .20, p=.42). In the sample excluding infants who were >40 months of age at the 36-month assessment, the standardised coefficient was β=.23 (95% CIs = .01 - .45, p=.05). The standardised coefficient between 12-month PAF and non-verbal ability slope was β=-.20 (95% CIs = -.57 - .17, p=.30).

*Verbal Ability*

In the full sample, the standardised coefficient between 12-month PAF and verbal ability intercept was β=.12 (95% CIs = -.05 - .30, p=.16). The standardised coefficient between 12-month PAF and non-verbal ability slope was β=-.04 (95% CIs = -.26 - .17, p=.70). In the sample excluding infants who were >40 months of age at the 36-month assessment, the standardised coefficient was β=.07 (95% CIs = -.11 - .26, p=.43). The standardised coefficient between 12-month PAF and verbal ability slope was β=-.03 (95% CIs = -.25 - .19, p=.78).
